# Perioperative Evaluation of Infective Endocarditis Via Multimodality Imaging for the Surgical Management of Aortic Root Abscess

**DOI:** 10.7759/cureus.17817

**Published:** 2021-09-08

**Authors:** Sudhakar Kinthala, Akhila Yarramneni, Jordan Huang, Natesh Yepuri, Poovendran Saththasivam, Sudhakar Sattur

**Affiliations:** 1 Anesthesiology, Guthrie Robert Packer Hospital, Sayre, USA; 2 Department of Surgery, Division of Cardiac Surgery, Guthrie Robert Packer Hospital, Sayre, USA; 3 Cardiology, Guthrie Robert Packer Hospital, Sayre, USA

**Keywords:** perioperative evaluation, infective endocarditis, multimodality imaging, surgical management, aortic root abscess

## Abstract

Infective endocarditis (IE) is an infection of the endothelium of the heart, that typically affects heart valves. While echocardiography remains crucial in the diagnosis and management of IE, multimodality cardiac imaging helps obtain additional information for the management of complex cases. Alternative imaging modalities such as computed tomography (CT), computed tomography angiography (CTA), and magnetic resonance imaging (MRI) are playing an increasing role in the diagnosis and management of IE, especially for patients with prosthetic valve endocarditis (PVE).

Here we present a case of a 60-year-old Caucasian male who was diagnosed with IE, complicated by aortic root abscess, and multiorgan failure. In this challenging case, multimodality cardiac imaging helped in the precise understanding of the extent of endocarditis, cannulation strategy, and direct the course of the surgical procedure that resulted in successful patient management.

## Introduction

Infective endocarditis (IE) has an annual incidence of 3-10/100,000 of the population with a mortality of up to 30% at 30 days [1]. IE can have a wide range of cardiac manifestations, including valvular vegetation, abscess, peri-annular extension of infection, and myopericarditis. IE can also involve other organs systems [2,3]. Echocardiography remains crucial in the diagnosis, and management of IE, however other imaging modalities, including cardiac computed tomography angiography (cardiac CTA), cardiac magnetic resonance imaging (CMR), and nuclear imaging, are helpful in obtaining additional information needed for the management of complex cases involving complications of the cardiac valves [4,5]. CTA, in particular, is helpful in determining the presence of perivalvular pathology such as abscesses, aneurysm, or pseudoaneurysm formation, particularly when aortic valve endocarditis or root abscess is suspected. It also provides additional value in preoperative planning and the evaluation of coronary anatomy and prosthetic valve function [3].

The presence of a paravalvular lesion on CT is a major criterion in the modified Duke criteria [5]. Decisions on surgical intervention are complex and multifactorial [6]. The presence of valve dysfunction causing heart failure, IE with fungi or drug-resistant organisms, and persistent bacteremia despite antimicrobial therapy are common indications for surgical management [2]. Aortic root abscess is a serious complication of aortic valve endocarditis, and is one of the indications for surgical intervention, with high perioperative morbidity, and mortality [2].

Here we present a case of IE, complicated by aortic root abscess and multiorgan failure, who underwent preoperative multimodality imaging using transthoracic echocardiography (TTE), and CTA of the chest. Multimodality imaging significantly helped with pre-surgical planning, and intraoperative transesophageal echocardiography (TEE) helped direct the course of the surgical procedure, resulting in successful surgical management. In this report, we discuss the value of perioperative multimodality imaging in the surgical management of aortic root abscess.

## Case presentation

A 60-year-old Caucasian man was transferred to our facility with a history of high fever, chills, fatigue, and shortness of breath. His past medical history was significant for diabetes mellitus, hypertension, severe aortic stenosis (AS), and atrial fibrillation (A-fib). 12-lead EKG showed A-fib with a rapid ventricular response. Blood culture was positive for methicillin-sensitive Staphylococcus aureus (MSSA).

**Figure 1:**
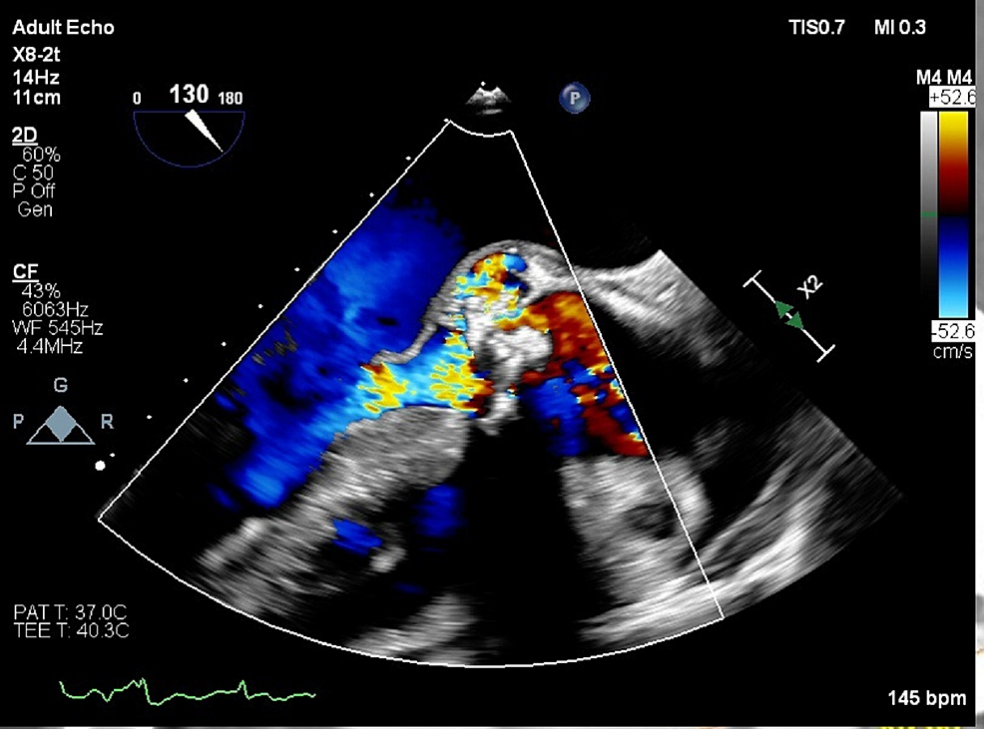
Pre-operative transesophageal echocardiogram. Mid-esophageal aortic valve long-axis view with color Doppler showing severe aortic regurgitation.

Upon admission, the patient was noted to have leukocytosis with a white blood cell count of 24 x 10^3^, renal insufficiency with a creatinine of 2.6 mg/dL, and oliguria. TEE showed tricuspid aortic valve with severe AS [Aortic Valve Area (AVA) 0.9 cm^2^], severe aortic insufficiency (AI) (Figure 1, Video 1), and aortic root abscess (Video 2) extending from the noncoronary cusp to the aorto-mitral curtain. No regional wall motion abnormality was found and the left ventricular ejection fraction was 60-65%.

**Video 1:** Pre-operative transesophageal echocardiogram. Mid-esophageal aortic valve long-axis view with color Doppler showing severe aortic regurgitation, and posterior root abscess.

**Video 2:** Pre-operative transesophageal echocardiogram. Mid-esophageal aortic valve long-axis view showing aortic valve abscess with leaflet perforation, and prolapse.

**Figure 2:**
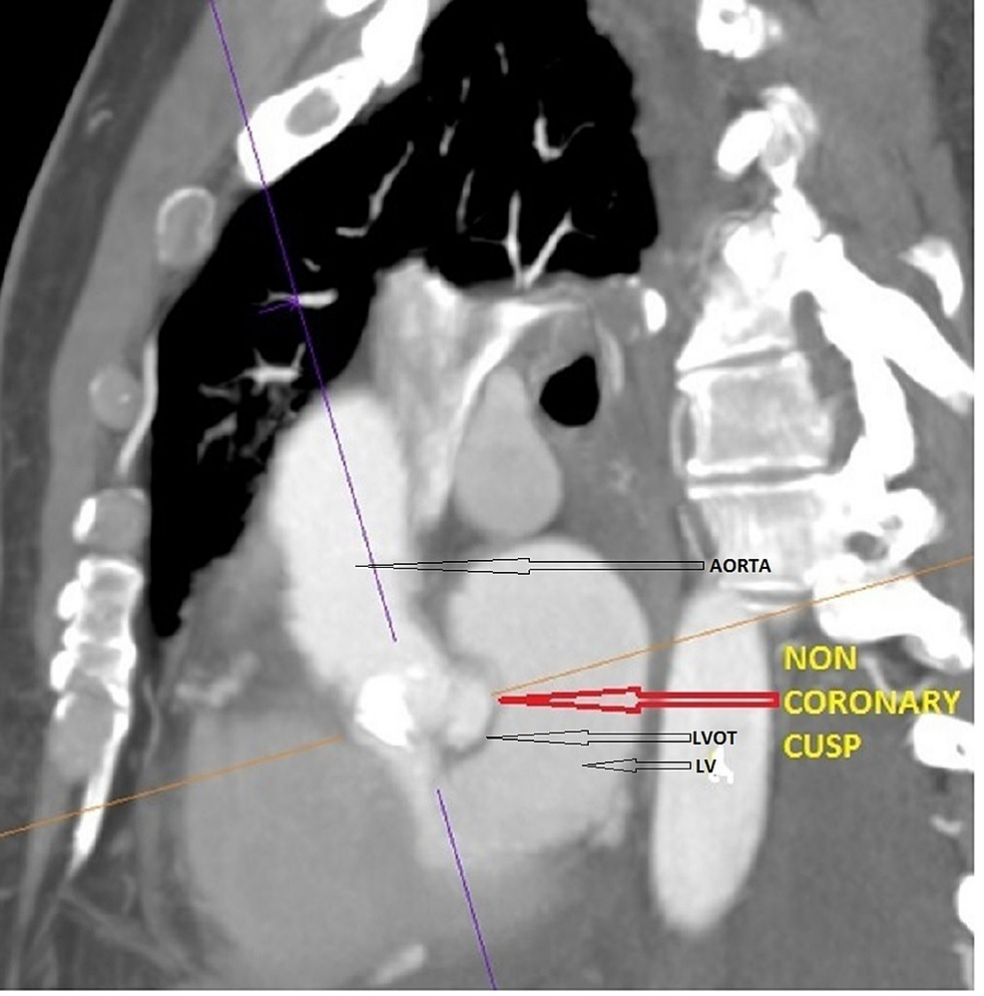
Computerized tomography of the chest (sagittal view) showing abscess of the non-coronary cusp. LV: Left Ventricle; LVOT: Left Ventricular Outflow Tract.

**Figure 3:**
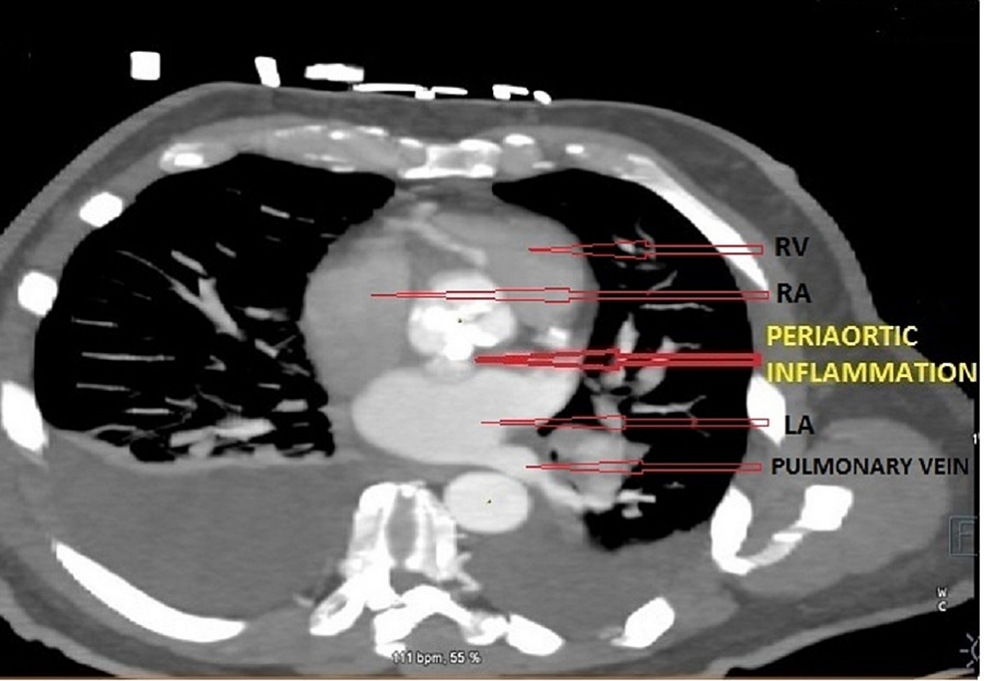
Computerized tomography of the chest (axial view) showing aortic abscess abutting left atrium. RV: Right Ventricle; RA: Right Atrium; LA: Left Atrium.

Cardiac CTA showed a large aortic root abscess cavity (Figures 2, 3), extensive bilateral renal and splenic infarcts, and bilateral pleural effusion. The patient was intubated in view of sepsis and multiorgan failure and started on norepinephrine for maintaining blood pressure. A decision was made to proceed with emergency surgical intervention.

A midline sternotomy was performed, and cardiopulmonary bypass was initiated. Aortotomy was performed. Upon direct inspection, vegetations were found on all cusps of the aortic valve, with very large vegetations on the noncoronary cusp. The noncoronary cusp was found to be separated from the annulus, and there was an abscess cavity extending from the annulus of the noncoronary cusp to the aorto-mitral curtain. The mitral valve was not involved.

**Figure 4:**
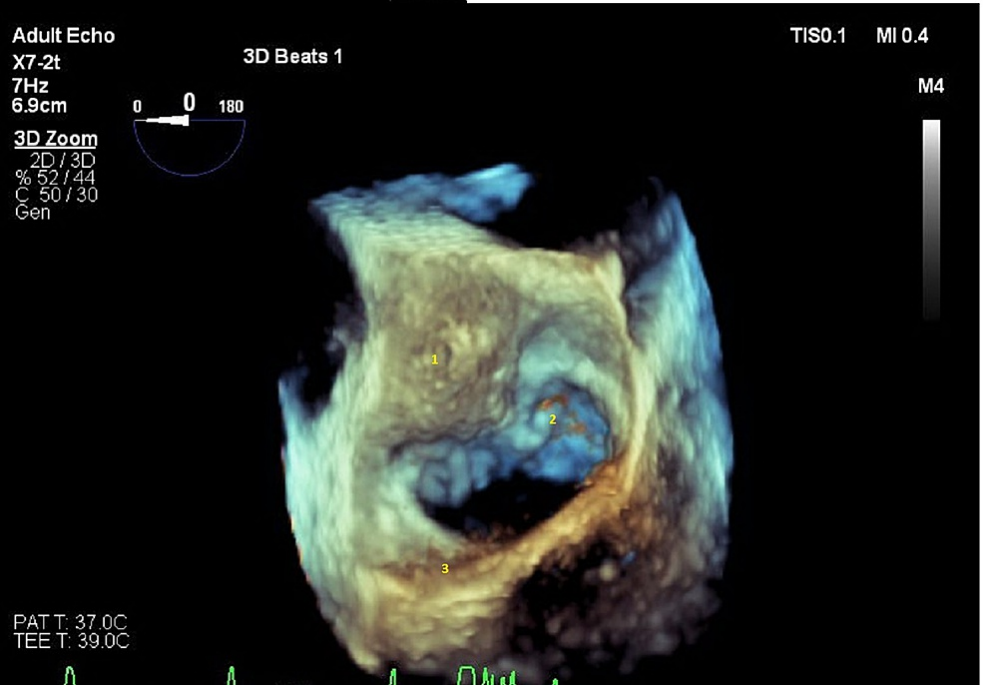
Three-dimensional transesophageal echocardiogram of left atrial En-face view of the mitral valve showing: 1. Bulging of aortic root abscess encroaching left atrium. 2. Anterior leaflet of the mitral valve. 3. Posterior leaflet of the mitral valve.

The intraoperative 3D TEE showed a questionable fibrinous exudate/vegetation on the roof of the left atrium in close proximity to the anterior annulus of the mitral valve. This finding was not obvious on the preoperative TEE. The fibrinous exudate/vegetation was difficult to visualize through the aortotomy, then a left atriotomy was performed, and vegetations were found on the roof of the left atrium. The abscess cavity below the noncoronary cusp was also found to be bulging toward the left atrial roof (Figure 4, Video 3); however, no erosion into the left atrium or fistula to the left atrium was found. The fibrinous exudate/vegetation was excised, the left atrium copiously irrigated, and the left atrium was closed. The aortic valve cusps were excised, and the abscess cavity was debrided and copiously irrigated. A patch repair of the abscess cavity was performed, and the annulus of the noncoronary aortic cusp was reconstructed using a bovine pericardial patch. A size 21 Edwards Magna Ease aortic valve (Edwards Lifesciences, Irvine, CA, USA) was then sutured to the aortic annulus. The remaining surgery was completed uneventfully, and the patient was transported to the intensive care unit for postoperative care.

**Video 3:** Intraoperative three-dimensional transesophageal echocardiogram. Left atrial En-face view of the mitral valve showing bulging of aortic root abscess encroaching left atrium.

The Gram stain of the fibrinous material/vegetation that was sent at the time of surgery was reported as positive for MSSA. The patient was weaned from inotropic support and mechanical ventilation. In view of renal failure, a left internal jugular vein tunneled dialysis catheter was placed. The patient had an uneventful post-operative course and was discharged home on aspirin, warfarin, furosemide, and intravenous cefazolin; on a three-month follow-up, the patient was found to be doing well with the return of renal function and discontinuation of dialysis.

## Discussion

IE has a broad spectrum of cardiac complications. Peri-annular extension and heart failure are common complications of IE [7,8]. Peri-annular infection is more commonly seen in aortic endocarditis than in endocarditis with mitral or tricuspid valve involvement. It can lead to uncontrolled infection [7]. It is important to identify perivalvular involvement in IE, because the presence of a perivalvular abscess significantly increases the risk of embolization, conduction abnormalities, and complete heart block, and is also associated with a poor prognosis [9,10].

Echocardiography plays an important role in the diagnosis of IE and the assessment of disease severity and therefore provides prognostic data such as embolic risk [11]. TTE may be similar to TEE in the evaluation of anterior structures of the heart, such as the tricuspid valve and right ventricular outflow tract. The sensitivity of TTE in detecting vegetations in patients with native valves is close to 70%, and because of shadowing from the structural components of the prosthetic valve, TTE has only 50% sensitivity in detecting vegetations of prosthetic valves [5]. TEE plays a significant role in detecting endocarditis and vegetations in patients with prosthetic valves as the sensitivity is more than 90% [2]. TEE allows for better evaluation of vegetations, and perivalvular abscesses than TTE [12]. The use of three-dimensional (3D) echocardiography provides added value to the evaluation of IE and complements conventional two-dimensional (2D) echocardiography due to improved spatial resolution. The 3D TEE allows for better delineation of valvular vegetations and abscesses, perforations and paravalvular leaks, dehiscence, and localization of infections with regards to valve anatomy and its relationship to surrounding cardiac structures [11].

In view of the aforementioned limitations of echocardiography, the 2015 European Society of Cardiology (ESC) IE guidelines include consideration for the use of cardiac CT in cases of possible IE or IE not confirmed by other criteria, with high clinical suspicion for both native and prosthetic-valve IE, and special emphasis on the detection of perivalvular complications [5]. When compared to intraoperative surgical findings, the CT scan has high sensitivity and specificity for diagnosing complications of endocarditis involving the aortic valve [5].

When compared to TEE, CT has an advantage in the detection of perivalvular extension of abscesses and pseudoaneurysms [13] and may provide more accurate anatomical information due to superior spatial resolution [14]. As CT can provide a means of noninvasive assessment of coronary artery disease, it helps in surgical planning [2]. Though the cardiac CT has multiple advantages, it is not superior to TEE at evaluating for valve perforation or the presence of an intracardiac fistula [13].

Small vegetations and valve leaflet perforations could potentially be missed on CT. Echocardiography does have the advantage of evaluating a hemodynamic function, so the two modalities should be used as complementary techniques [15]. Though CT has the risks of radiation exposure and contrast-induced renal injury, there is the potential advantage of providing relevant diagnostic information. Because of its capability in imaging the entire body, CT also allows the detection of extracardiac IE-related lesions [2]. Due to the dynamic nature of IE with changing vegetation sizes, and the potential to rapidly develop local complications, TEE becomes vital for intraoperative assessment. It is reasonable to state that cardiac CT complements echocardiography for evaluating and diagnosing IE and its complications [2].

Fluorodeoxyglucose positron emission tomography (FDG-PET) is useful workup to identify nidus of infection when infective endocarditis is suspected in patients with prosthetic valve or cardiac implanted electronic devices with negative or inconclusive echocardiography and CT. Cardiac MRI could be considered to quantify valvular regurgitation in patients with poor echocardiography and to assess the intracardiac spread of disease in a patient who has a contraindication to receive contrast [16].

In our case, perioperative imaging played a significant role in surgical decision-making. 2D TEE showed severe AI and possible separation of the noncoronary cusp from the annulus, an abscess cavity in the area of the noncoronary cusp, and no endocarditis of the mitral valve. To delineate the abscess cavity and rule out a fistula between the left atrium and aorta, a CTA chest was performed, which showed an abscess cavity and no obvious fistula tract between the left atrium and aorta.

The CTA showed that the periaortic inflammation extended up to the roof of the left atrium, but no fistula tract was visible between the aorta and left atrium. The presence of aortic root abscess was also confirmed by preoperative 2D TEE. However, the presence of fibrinous material on the roof of the left atrium, which was discovered on the intraoperative 3D TEE and prompted left atriotomy with excision of that material, was not present on preoperative 2D TEE imaging. Intraoperative 3D TEE was therefore crucial in finding the fibrinous material and making the appropriate intraoperative surgical decision, which resulted in the patient's successful outcome. Missing the fibrinous material/vegetation might have resulted in continued positive blood cultures, with increased risk of subsequent recurrence of endocarditis, therefore potentially defeating the purpose of the surgical intervention.

## Conclusions

Multimodality imaging is paramount in the management of complex cases of infective endocarditis, which is clearly illustrated in our patient. Multimodality imaging helps with the precise understanding of the extent of endocarditis and aids in directing appropriate surgical intervention for favorable patient outcomes.
